# ^18^F-FDG PET/MRI Multiparametric Imaging Features for Predicting MYCN Amplification Status in Children With High-Risk Neuroblastoma

**DOI:** 10.31083/RN51030

**Published:** 2026-06-26

**Authors:** Jiangtao Liang, Feng Li, Zhongxiang Ding, Bing Yan, Yanfang Zhu, Fangxiao Wang, Weiling Xuan

**Affiliations:** ^1^Hangzhou Universal Medical Imaging Diagnostic Center, 310009 Hangzhou, Zhejiang, China; ^2^Department of Radiology, Affiliated Hangzhou First People’s Hospital, Westlake University School of Medicine, 310006 Hangzhou, Zhejiang, China; ^3^Hangzhou Xixi Hospital Affiliated to Zhejiang Chinese Medical University, 310023 Hangzhou, Zhejiang, China

**Keywords:** neuroblastoma, *MYCN* amplification, positron emission tomography, magnetic resonance imaging

## Abstract

**Background:**

This study aimed to explore the value of multiparameter imaging features of ^18^F-fluorodeoxyglucose positron emission tomography/magnetic resonance imaging (^18^F-FDG PET/MRI) in predicting *MYCN* amplification status in children with high-risk neuroblastoma (HR-NB) and to construct a combined predictive model.

**Methods:**

A retrospective analysis of the clinical and PET/MRI imaging data of 121 children with HR-NB who were examined at our center between December 2018 and December 2025. According to the tumor *MYCN* amplification status, patients were divided into amplification (MNA group) and non-amplified (MYCN-NA group) groups. The differences in clinical characteristics and PET/MRI parameters between the two groups were also compared. Multivariate logistic regression analysis was used to identify the independent predictive factors. The predictive performance of each parameter and the combined model was evaluated using receiver operating characteristic (ROC) curves, calibration curves, and decision curves. The combined model was visualized using nomograms.

**Results:**

The imaging-defined risk factor (IDRF) positivity rate, proportion of stage M cases, and detection rates of tumor necrosis and hemorrhage were significantly higher in the MNA group than in the MYCN-NA group (all *p* < 0.05). The MNA group demonstrated a significantly larger mean diameter, higher maximum standardized uptake value (SUV_max)_, mean standardized uptake value (SUV_mean_), metabolic tumor volume (MTV), and total lesion glycolysis (TLG), and a significantly lower mean apparent diffusion coefficient (ADC_mean_) (all *p* < 0.001). Multivariate regression analysis showed that SUV_max_ (odds ratio (OR) = 1.71, 95% CI: 1.02–2.86, *p *= 0.042), MTV (OR = 1.075, 95% CI: 1.009–1.145, *p *= 0.026), and ADC_mean _(OR = 0.34, 95% CI: 0.16–0.72, *p *= 0.004) were independent predictors of *MYCN* amplification. The combined model predicted *MYCN* amplification with an AUC of 0.916 (95% CI: 0.867–0.965), sensitivity of 83.3%, and specificity of 81.0%, outperforming all single parameters. Calibration and decision curve analyses indicated good calibration and clinical utility of the combined model.

**Conclusion:**

The predictive model constructed by combining SUV_max_, MTV, and ADC_mean_ can noninvasively distinguish *MYCN* amplification status in HR-NB patients.

## 1. Introduction

Neuroblastoma (NB) is the most common extracranial solid tumor in children, originating from primitive neural crest cells of the sympathetic nervous system [1]. It exhibits great clinical and biological heterogeneity, with manifestations ranging from spontaneous regression to highly aggressive, widely metastatic malignant disease with a poor prognosis [2]. *MYCN* gene amplification (MNA) occurs in approximately 20% of patients with NB and is one of the most important molecular biological markers closely associated with high tumor aggressiveness and poor prognosis [3]. It is recognized as an independent prognostic factor by the International Neuroblastoma Risk Group (INRG) classification system [4]. However, the spatial heterogeneity of *MYCN* and the occurrence of low-level copy number gain (*MYCN* gain) increase the difficulty of accurate assessment [5]. Although fluorescence in situ hybridization (FISH) is the gold standard for detecting *MYCN* amplification, its invasiveness, high cost, and sample limitations restrict its widespread clinical application [6]. Therefore, noninvasive, accurate, and real-time detection technologies must be developed.

^18^F-fluorodeoxyglucose positron emission tomography/computed tomography (^18^F-FDG PET/CT), a noninvasive molecular imaging technique that reflects the glucose metabolic activity of tumor cells, has been widely used in the diagnosis, staging, and prognostic assessment of NB [7]. The study has shown that PET/CT metabolic parameters, such as the maximum standardized uptake value (SUV_max_), metabolic tumor volume (MTV), and total lesion glycolysis (TLG), are closely associated with the *MYCN* amplification status and patient prognosis [8]. In addition, PET/CT radiomics can extract high-throughput imaging features reflecting tumor heterogeneity and biological characteristics, showing its potential in predicting *MYCN* amplification and risk stratification in NB [9]. PET/magnetic resonance imaging (MRI) combines the functional metabolic information of PET with the high soft tissue resolution of MRI, offering superior soft tissue contrast and integrated functional information. It can provide more comprehensive multiparameter imaging features of tumors and reveal tumor tissue heterogeneity and biological activity at the voxel level through analysis [10], thus potentially further improving the accuracy of *MYCN* amplification status prediction in children with high-risk neuroblastoma [11].

This study integrated ^18^F-FDG PET/MRI-based imaging parameters and clinical data to construct a multimodal imaging model to assess the noninvasive predictive capacity for *MYCN* gene amplification status in high-risk neuroblastoma (HR-NB) patients.

## 2. Materials and Methods

### 2.1 Study Subjects

A retrospective analysis was conducted on the clinical and PET/MRI imaging data of 121 children with HR-NB who underwent examinations at our center between December 2018 and December 2025. Patients were categorized according to the INRG classification system [4] as follows: All children underwent comprehensive PET/MRI examinations before treatment and were confirmed to have HR-NB through histopathological and gene-related tests. The inclusion criteria were as follows: (1) Pathologically confirmed neuroblastoma; (2) Newly identified lesions without any prior antitumor treatment; (3) Clear, full-body PET/MRI images available before treatment; and (4) Underwent PET/MRI examination 40–60 min after injection of the imaging agent. Exclusion criteria: (1) PET or MRI images did not meet diagnostic standards (e.g., with metal or motion artifacts, etc.); (2) Suspected deviation in SUV values; (3) Patients with additional systemic malignant tumors; (4) Patients who had received any form of treatment (e.g., radiotherapy, chemotherapy, etc.) before PET/MRI examination. The imaging data collected included tumor necrosis, calcification, hemorrhage, mean diameter, SUV_max_, mean standardized uptake value (SUV_mean_), peak standardized uptake value (SUV_peak_), MTV, TLG, mean apparent diffusion coefficient (ADC_mean_), and coefficient of variation (CV). The clinical data included age, sex, primary tumor site, imaging-defined risk factors (IDRFs), and INRG stage. The International Neuroblastoma Risk Group Staging System (INRGSS) and International Neuroblastoma Pathological Classification System were used to determine the staging and histological types, respectively.

Based on the *MYCN* amplification status, patients were divided into *MYCN* amplification (MNA) and non-amplified (MYCN-NA) groups. 

The reporting of this study follows the STROBE guidelines (cohort design). A completed checklist is available in **Supplementary File 1**.

### 2.2 Instruments and Equipment

Imaging data were acquired using a GE Healthcare integrated time-of-flight (TOF) PET/MRI system (GE SIGNA, Milwaukee, WI, USA). This system consists of a PET detector equipped with TOF technology and a latest-generation 750W 3.0T MRI device. The TOF-PET detector utilizes advanced solid-state silicon photomultipliers (SiPMs) and the latest Lutetium Yttrium Orthosilicate (LYSO) crystals. The radiopharmaceutical used was ^18^F-FDG. MRI scans were performed as plain scans only, with scanning sequences including axial T1-weighted imaging (T_1_WI), axial T2-weighted imaging with fat suppression (T_2_WI-FS), and axial Diffusion-Weighted Imaging (DWI) (b = 800 s/mm^2^). The slice thickness was 6 mm, and the interslice gap was 2 mm, using parallel acquisition techniques and a dedicated phased array coil to receive signals. The scan range was from the vertex to the mid-femur of the participants.

### 2.3 Data Analysis and Measurement

All images were interpreted by three radiologists with more than ten years of experience in pediatric tumor diagnosis and certification in both MRI and nuclear medicine (one attending physician and two chief physicians). A consensus was reached through joint discussion, and in the case of disagreement, a consensus was reached after further discussion. Quantitative parameters were measured using the PET VCAR software (version 4.6, GE Healthcare). Image analysis was primarily based on visual and semi-quantitative analyses of the images.

The fixed-threshold method was used to delineate the region of interest, with a threshold set at 40% of the SUV_max_. The PET metabolic parameters of the lesions (SUV_max_, SUV_mean_, SUV_peak_, MTV, and TLG) were automatically obtained. In the ADC images, the region with the highest tumor metabolism on PET was selected as the reference. On three consecutive slices, an region of interest (ROI) with a diameter >5 mm was manually drawn, avoiding areas of calcification, hemorrhage, cystic change, or tumor necrosis. The mean ADC of the three ROIs was used as the final ADC _mean_ (×10^–3^ mm^2^/s) for the statistical analysis. The CV value was calculated as follows:

CV value = standard deviation of SUV / SUV_mean_ × 100%

The criteria for tumor necrosis were as follows: a metabolic defect area within the tumor on the PET sequence, a high signal on the T_2_WI sequence, and a low signal on both the T_1_WI and DWI sequences indicated the presence of tumor necrosis.

### 2.4 MYCN Gene Amplification Detection

The *MYCN* amplification status was determined by FISH of formalin-fixed paraffin-embedded samples obtained from biopsy or surgical resection. According to the international consensus on the molecular diagnosis of neuroblastoma, if the copy number of the target gene equals the copy number of chromosome 2, that is, ≤2, it is considered negative; a copy number of 3–9 is considered gained; and a copy number five times or more than the copy number of chromosome 2, that is, ≥10, is considered amplified [3].

### 2.5 Statistical Analysis

SPSS software V23.0 (IBM Corp., Armonk, NY, USA) and R software V4.2.3 (R Foundation for Statistical Computing, Vienna, Austria) were used for data processing and statistical analyses. Continuous variables were compared using the independent-sample *t* test (normally distributed) or Mann-Whitney U test (non-normally distributed). Categorical variables were compared using the chi-square or Fisher’s exact test. Variables with *p* < 0.05 in univariate analysis were entered into multivariate logistic regression using forward stepwise selection (likelihood ratio test). Model fit was assessed by the Hosmer-Lemeshow test (*p* > 0.05 indicated good fit). Receiver operating characteristic (ROC) curves were used to evaluate diagnostic performance, with calculation of AUC, sensitivity, and specificity. Optimal cut-offs were determined by the Youden index. Calibration curves and decision curve analysis (DCA) were used to assess model calibration and clinical utility. A nomogram was constructed to visualize the combined model.

### 2.6 Reproducibility Assessment

Three radiologists independently reviewed all the images. For qualitative features, such as tumor necrosis, calcification, and hemorrhage, inter-observer agreement was determined using the Kappa statistic, with a value of ≥0.75 indicating reliable consistency. To examine the potential variability arising from manual segmentation and ROI placement, the intraclass correlation coefficient (ICC) was computed for all quantitative parameters, including mean diameter, SUV_max_, SUV_mean_, MTV, and ADC_mean_ based on the measurements from the three observers. An ICC exceeding 0.75 was considered to indicate excellent agreement.

## 3. Results

### 3.1 Clinical Features

Among the 121 HR-NB patients, there were 49 (40.5%) and 72 (59.5%) in the MNA and MYCN-NA groups, respectively. Of these, 69 were male (57%) and 52 were female (43%), with no statistically significant difference between the groups (χ^2^ = 1.311, *p* = 0.252). The mean ages of the two groups were 3.03 ± 1.74 years and 3.28 ± 2.06 years, respectively, with no statistically significant difference (*t* = –0.95, *p* = 0.344). Tumors were located in the adrenal gland in 90 cases (74.4%) and in other regions in 31 cases (cervical sympathetic ganglia, 3 cases; mediastinum, 9 cases; retroperitoneal sympathetic chain, 12 cases; presacral pelvic region, 7 cases). There was no statistically significant difference between the two groups (χ^2^ = 1.169, *p* = 0.280). The proportion of patients with IDRFs was significantly higher in the MNA group than in the MYCN-NA group (93.9% vs. 79.2%, χ^2^ = 4.994, *p* = 0.025). Regarding INRG staging, both groups were predominantly in the M stage, with the MNA group accounting for 87.8% and the MYCN-NA group accounting for 70.8% of the patients. The difference in stage distribution between the two groups was significant (χ^2^ = 4.808, *p* = 0.028) (Table 1).

**Table 1.** **Clinical characteristics and PET/MRI qualitative features with significant differences between children with neuroblastoma in the MNA and MYCN-NA groups**.

Group	n	IDRFs (n)		INRG stage (n)				Necrosis (n)		Hemorrhage (n)	
		Positive	Negative	L1	L2	M	Ms	Yes	No	Yes	No
MNA	49	46	3	0	6	43	0	31	18	20	29
MYCN-NA	72	57	15	6	15	51	0	26	46	14	58
χ^2^ Value		4.994		4.808				8.620		6.590	
*p * Value		0.025		0.028				0.003		0.010	

Note: The statistical method used was the chi-square test. PET, positron emission tomography; MRI, magnetic resonance imaging; MNA, *MYCN* amplification; MYCN-NA, *MYCN* non-amplified; IDRFs, imaging-defined risk factors; INRG, International Neuroblastoma Risk Group.

### 3.2 Comparison of ^18^F-FDG PET/MRI Imaging Parameters

The detection rate of tumor necrosis in the MNA group (63.3%) was higher than that in the MYCN-NA group (36.1%), and this difference was statistically significant (χ^2^ = 8.62, *p* = 0.003). Signs of hemorrhage were also more common in the MNA group (40.8% vs. 19.4%, χ^2^ = 6.59, *p* = 0.010), as shown in Table 1. The difference in the detection rate of calcification between the two groups was not statistically significant (73.5% vs. 65.3%, χ^2^ = 0.91, *p* = 0.340).

The average tumor diameter, SUV_max_, SUV_mean_, MTV, and TLG were all significantly higher in the MNA group compared to the MYCN-NA group, with statistically significant differences (Z = –4.15 to 4.23, all *p* < 0.001). In contrast, the ADC_mean_ was significantly lower in the MNA group than in the MYCN-NA group (Z = 4.23, *p* < 0.001) (Table 2). The difference in the CV between the two groups was not statistically significant (Z = –0.87, *p* = 0.384).

**Table 2.** **Quantitative PET/MRI features with significant differences between children with neuroblastoma in the MNA and MYCN-NA groups**.

Group	Mean diameter (cm)	SUV_max_	SUV_mean_	MTV (cm^3^)	TLG (g)	ADC_mean _(×10^–3^mm^2^/s)
MNA	7.0 (5.3, 9.0)	7.5 (5.5, 9.5)	4.1 (3.1, 5.1)	115.0 (57.2, 197.0)	528.0 (215.0, 1037.7)	0.45 (0.38, 0.71)
MYCN-NA	5.5 (3.9, 7.3)	4.6 (3.5, 7.0)	2.6 (1.9, 3.7)	45.0 (19.0, 97.5)	143.7 (60.6, 326.5)	0.73 (0.60, 0.80)
*Z *value	–3.21	–4.15	–4.02	–3.85	–3.92	4.23
*p* value	<0.001	<0.001	<0.001	<0.001	<0.001	<0.001

Note: The statistical method used was the U test. SUV, standardized uptake value; MTV, metabolic tumor volume; TLG, total lesion glycolysis; ADC, Apparent Diffusion Coefficient.

### 3.3 Logistic Regression Analysis Combining Clinical and Imaging Characteristics

**Fig. 1.**
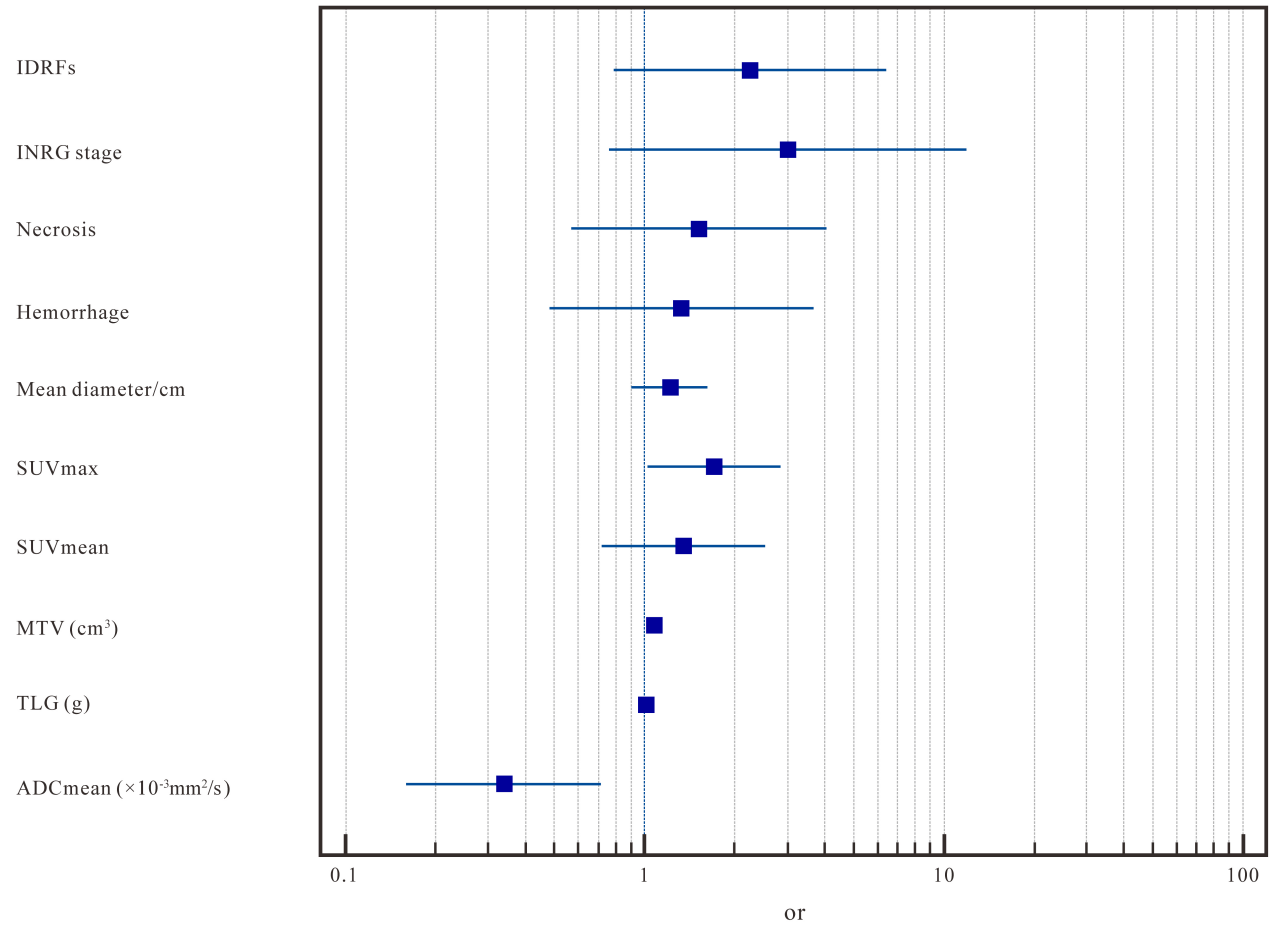
**Forest plot of multivariate logistic regression analysis based on clinical features and PET/MRI imaging characteristics**.

Variables with statistically significant differences (IDRFs, INRG stage, tumor necrosis, hemorrhage, mean diameter, SUV_max_, SUV_mean_, MTV, TLG, and ADC_mean_) were included in the multivariate logistic regression analysis. The analysis showed that SUV_max_ (OR = 1.71, 95% CI: 1.02–2.86, *p* = 0.042), MTV (OR = 1.075, 95% CI: 1.009–1.145, *p* = 0.026), and ADC_mean_ (OR = 0.34, 95% CI: 0.16–0.72, *p* = 0.004) were independent predictors of *MYCN* amplification. The Hosmer-Lemeshow test indicated good model fit (χ^2^ = 6.732, *p* = 0.565). Fig. 1 shows a forest plot of each variable in the multivariate logistic regression analysis.

### 3.4 Evaluation of Model Predictive Performance

**Fig. 2.**
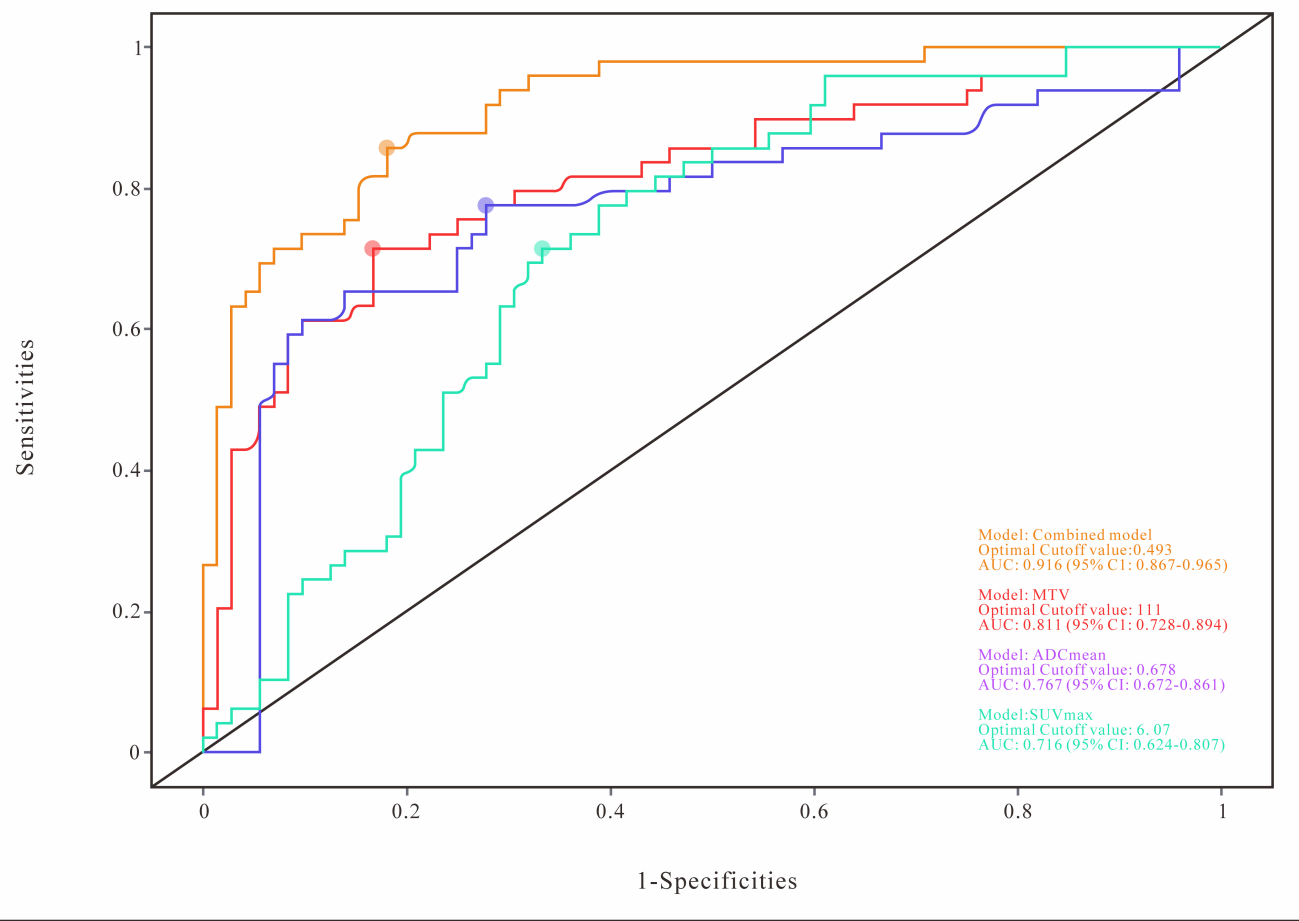
**Comparison of ROC curves for the four models**. Black solid diagonal line: chance performance reference (AUC = 0.5). ROC, receiver operating characteristic.

**Fig. 3.**
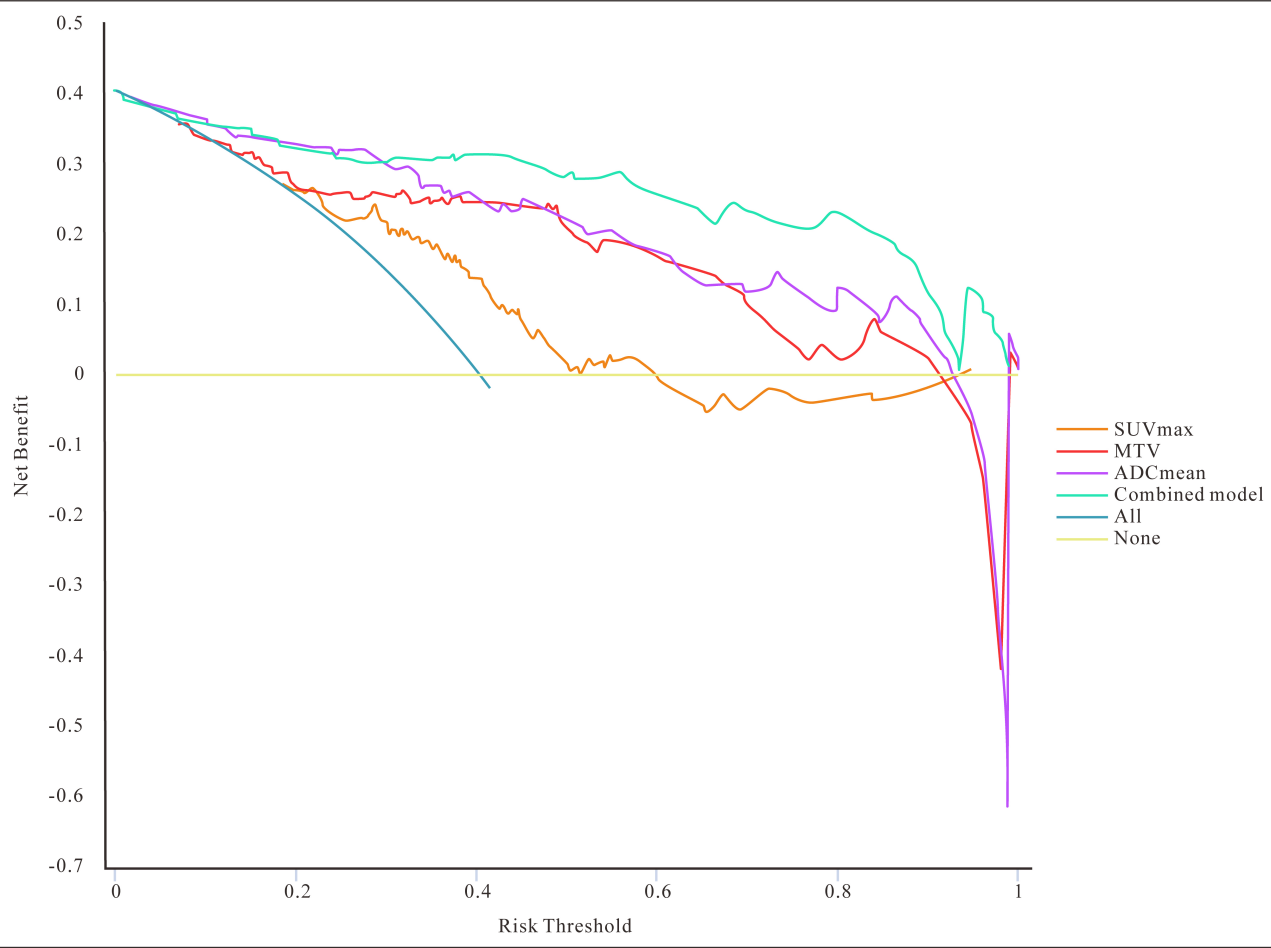
**Comparison of the DCA curves of the four models**. The horizontal axis represents the diagnostic threshold, and the vertical axis represents the net benefit of the model at that threshold. DCA, decision curve analysis.

**Fig. 4.**
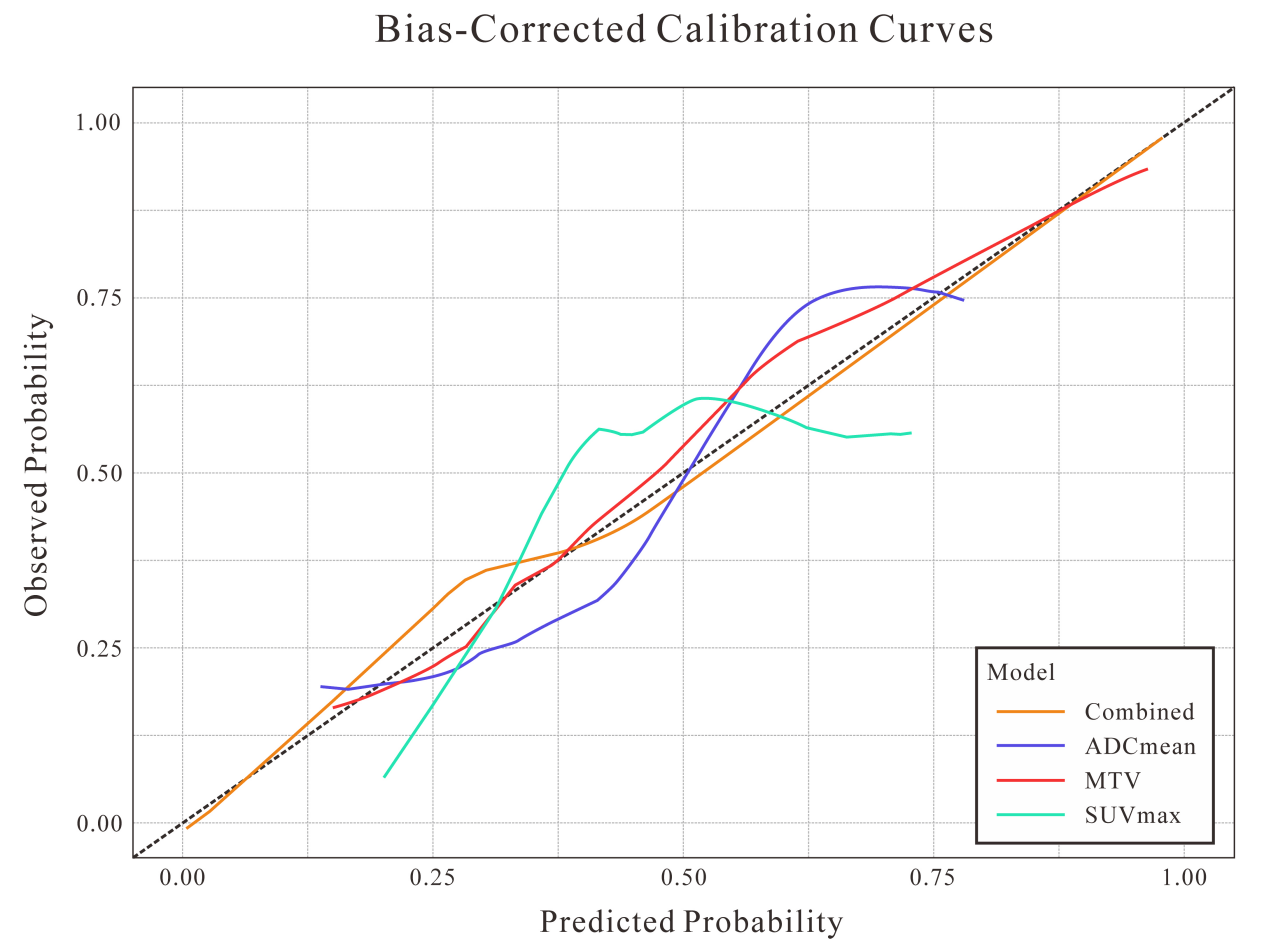
**Calibration curves of four models**. The horizontal axis represents the probability predicted by the model, and the vertical axis represents the actual observed probability. The black dashed diagonal line represents perfect calibration (ideal reference), where predicted probability equals observed probability.

**Fig. 5.**
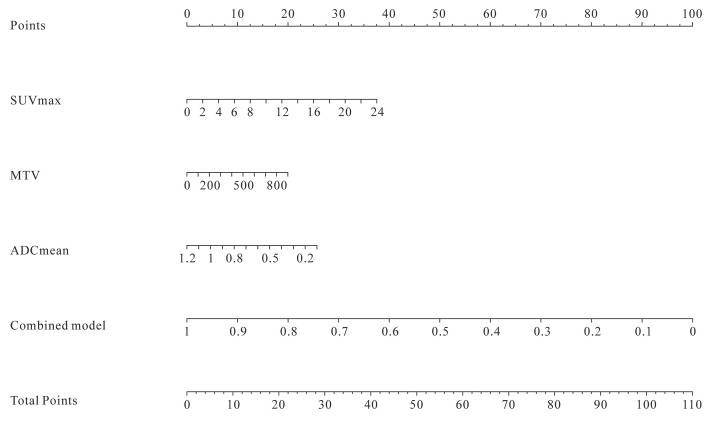
**Nomogram of the combined model**.

ROC curve analysis (Fig. 2) indicated that the independent predictors SUV_max_ (AUC: 0.716, 95% CI: 0.624–0.807, sensitivity: 0.683, specificity: 0.714), MTV (AUC: 0.811, 95% CI: 0.728–0.894, sensitivity: 0.750, specificity: 0.762), and ADC_mean_ (AUC: 0.767, 95% CI: 0.672–0.861, sensitivity: 0.667, specificity: 0.667) all demonstrated relatively high diagnostic values for *MYCN* amplification among the relevant parameters. By integrating the three independent predictors into a combined predictive model, the diagnostic performance was further enhanced, with an AUC of 0.916 (95% CI: 0.867–0.965, sensitivity: 0.833, specificity: 0.810) (Table 3). The DCA curves of the four models (Fig. 3) suggest that the combined model provides the best clinical utility. All four models were evaluated using calibration curves (Fig. 4). The nomogram of the combined model is presented in Fig. 5.

**Table 3.** **Diagnostic efficiency of important parameters in differentiating between the MNA and MYCN-NA groups**.

Variable	Cut-off	AUC (95% CI)	Sensitivity (95% CI)	Specificity (95% CI)	*p* value
SUV_max_	6.07	0.716 (0.624–0.807)	0.683 (0.566–0.785)	0.714 (0.607–0.806)	<0.001
MTV (cm^3^)	111	0.811 (0.728–0.894)	0.750 (0.636–0.842)	0.762 (0.658–0.848)	<0.001
ADC_mean _(×10^–3^mm^2^/s)	0.678	0.767 (0.672–0.861)	0.667 (0.549–0.770)	0.667 (0.557–0.765)	<0.001
Combined model	-	0.916 (0.867–0.965)	0.833 (0.727–0.912)	0.810 (0.711–0.888)	<0.001

### 3.5 Consistency Test

The Kappa coefficients for necrosis, calcification, and hemorrhage were 0.802 (95% CI: 0.695–0.908), 0.719 (95% CI: 0.593–0.845), and 0.735 (95% CI: 0.614–0.856), respectively. The overall concordance rates were 90.1%, 86.0%, and 86.8%, respectively, suggesting a high level of inter-observer agreement (all *p* < 0.001; Table 4).

**Table 4.** **Consistency check results of qualitative features**.

Variable	n (% of concordance)	Kappa (95% CI)	*p* value
Necrosis	109/121 (90.1%)	0.802 (0.695–0.908)	<0.001
Calcification	104/121 (86.0%)	0.719 (0.593–0.845)	<0.001
Hemorrhage	105/121 (86.8%)	0.735 (0.614–0.856)	<0.001

For quantitative parameters, such as mean diameter, SUV_max_, SUV_mean_, MTV, and ADC_mean_, the ICC was computed based on the measurements obtained from the three observers. The ICC values were 0.851 (95% CI: 0.805–0.889), 0.969 (95% CI: 0.958–0.977), 0.918 (95% CI: 0.892–0.939), 0.951 (95% CI: 0.936–0.963), and 0.869 (95% CI: 0.827–0.903), respectively, all of which exceeded 0.75, indicating excellent reliability in the measurements of these quantitative parameters (all *p* < 0.001; Table 5).

**Table 5.** **Consistency check results of quantitative features**.

Variable	ICC (95% CI)	*p* value
Mean diameter (cm)	0.851 (0.805–0.889)	<0.001
SUV_max_	0.969 (0.958–0.977)	<0.001
SUV_mean_	0.918 (0.892–0.939)	<0.001
MTV	0.951 (0.936–0.963)	<0.001
ADC_mean_	0.869 (0.827–0.903)	<0.001

ICC, intraclass correlation coefficient.

## 4. Discussion

*MYCN* gene amplification is a major driver of oncogenic events in children with neuroblastoma, occurring in approximately 20%–30% of patients with HR-NB, and plays a crucial role in its onset and early development [12]. This study systematically evaluated the noninvasive predictive value of ^18^F-FDG PET/MRI multiparametric imaging features combined with clinical indicators of *MYCN* gene amplification status in children with HR-NB. The results showed that SUV_max_, MTV, and ADC_mean _were independent predictors of *MYCN* amplification, and the combined model achieved an AUC of 0.916, with a sensitivity of 0.833 and specificity of 0.810. This was significantly superior to the single-parameter models, reflecting the potential of multimodal imaging integration for molecular subtyping. This highlights the unique advantages of PET/MRI multimodal imaging in revealing tumor metabolic activity and tissue microenvironment heterogeneity, thereby providing important imaging evidence for precise risk stratification and individualized treatment [13].

The results of this study showed that the detection rates of tumor necrosis and hemorrhage in the MNA group were significantly higher than those in the MYCN-NA group, indicating that tumors with *MYCN* amplification exhibit greater aggressiveness and heterogeneity. This is consistent with Campbell et al.’s [14] view regarding the association between *MYCN* amplification and high tumor aggressiveness and further substantiates the biological basis of *MYCN* amplification as a marker of poor prognosis [15]. These findings echo previous research regarding the molecular mechanisms underlying the high proliferative activity, necrosis-prone nature, and invasiveness of tumors with *MYCN* amplification, reinforcing the intrinsic link between imaging features and molecular biological characteristics and confirming the value of qualitative imaging features as reflections of biological aggressiveness [16].

This study found that SUV_max _was significantly positively correlated with *MYCN* amplification, supporting the conclusion of Hu et al.’s [17] systematic review that a high SUV_max_ indicates a poor prognosis. This finding is highly consistent with the reports by Li et al. [18] and Liu et al. [19] regarding the important role of SUV_max _in predicting *MYCN* amplification and patient prognosis, further reinforcing the reliability and clinical significance of SUV_max _as an indicator of tumor metabolic activity in neuroblastoma. In this study, MTV and TLG were significantly elevated in the MNA group, echoing the PET/CT-based findings of Li et al. [18] and Feng et al. [20], which are closely related to the aggressive and rapidly proliferative biological behavior induced by *MYCN* amplification [21]. However, this differs from Hu et al.’s [17] meta-analysis, in which the statistical significance of MTV and TLG in prognostic prediction was not observed. This discrepancy may stem from our study’s use of PET/MRI fusion technology, which, by combining the high soft tissue resolution and functional information of MRI, enhances the ability to reflect tumor volume and metabolic heterogeneity, showcasing the advantage of PET/MRI in capturing complex tumor biological features [22]. Furthermore, a significant decrease in ADC_mean _suggests that *MYCN*-amplified tumors have higher cellular density and reduced extracellular space, limiting water molecule diffusion, which reflects the sensitivity of PET/MRI DWI parameters to microenvironmental and tissue structural changes [23]. There have been few previous reports on the relationship between ADC and *MYCN* amplification; our study adds evidence to this area, emphasizing that multimodal imaging joint analysis provides a new biological dimension for molecular phenotype prediction in tumors, improving the accuracy and stability of predictive models. PET/MRI examinations have expanded the application of traditional single-parameter PET/CT [7]. Changes in these imaging parameters not only reveal the metabolic and microstructural characteristics of *MYCN*-amplified tumors but also provide reliable biomarkers for noninvasive predictions. SUVmax, MTV, and ADCmean are independent predictive factors of *MYCN* amplification status in HR-NB children [24]. The combined model, which integrates metabolic activity (SUV_max_), tumor volume (MTV), and tissue structure (ADC_mean_), significantly improved the accuracy and clinical utility of predictions, performing outstandingly in the ROC curve, calibration curve, and decision curve analysis, demonstrating the potential of multiparametric imaging features in precision medicine [25]. This advantage has also been reflected in previous studies through the construction of multi-omics parameter models, showing that integrating multidimensional information helps improve the accuracy of identifying *MYCN* copy number categories and suggests future research directions for multimodal, multiparameter joint analysis [26].

The limitations of this study primarily include the following aspects: (1) Limited sample size: This study retrospectively analyzed 121 children with high-risk neuroblastoma. Although the sample size was sufficient to ensure the validity of the statistical analyses to a certain extent, it still fell within a medium-scale range. The insufficient sample size may affect the model’s generalizability and stability [27]. (2) Limitations of the research methodology: This study used a retrospective design, which is subject to selection and information biases. In addition, the interpretation of certain imaging features depends on the subjective judgment of experienced physicians. Although consistency tests were conducted to reduce subjective errors, they cannot be completely avoided [28]. (3) All cases were collected from our center, which may introduce biases due to regional and equipment differences, affecting the external applicability of the model [29]. (4) Lack of in-depth validation of molecular mechanisms: Although this study explored the association between imaging parameters and *MYCN *amplification status, it did not further investigate the direct relationship between imaging features and molecular biological mechanisms [30].

Although the combined predictive model constructed in this study demonstrated good diagnostic value, it still has limitations, such as a limited sample size, single-center design, and insufficient integration of multi-omics data. Future studies should focus on the following directions: (1) Conducting multicenter, large-sample prospective studies to validate and enhance the model’s generalizability and stability; (2) Incorporating automated radiomics and machine learning technologies to improve the objectivity of feature extraction and predictive accuracy, thus promoting intelligent modeling [9]; (3) Integrating multi-omics data, such as genomics and proteomics, to enhance the biological interpretability of the model [26]; (4) Combining more clinical and pathological indicators to build a comprehensive risk stratification and personalized treatment support system. Advancements in these directions will promote the clinical application and deeper scientific exploration of this model for the noninvasive prediction of *MYCN* amplification status in high-risk neuroblastoma.

## 5. Conclusion

This study fills the gap in the use of multiparametric ^18^F-FDG PET/MRI imaging to predict *MYCN* amplification in high-risk neuroblastoma patients. This is the first study to systematically integrate PET metabolic parameters and MRI functional imaging features to establish a combined predictive model that includes SUV_max_, MTV, and ADC_mean_, which significantly improved predictive accuracy and clinical utility. This model provides an effective tool for noninvasive, real-time, and cost-effective auxiliary diagnosis, aiding in the early identification of high-risk patients, guiding personalized treatment, and reducing reliance on invasive testing. The research methodology is rigorous, and the results are reliable, laying a solid foundation for subsequent multicenter and multi-omics integration studies and advancing the development of precise risk assessment and molecular imaging diagnosis of high-risk neuroblastoma.

## Data Availability

The data and materials are available from the corresponding author upon request.
