# Supplementary material for: 18F-FDG PET/MRI Multiparametric Imaging Features for Predicting MYCN Amplification Status in Children With High-Risk Neuroblastoma
Source: Rev Neurol. 2026 Jun 26;81(6):51030. doi: 10.31083/RN51030 (PMC13339796; doi:10.31083/RN51030)
Supplement: Supplementary file 1 [file 1576-6578-81-6-51030-s1.zip › STROBE_checklist.docx]

STROBE Statement—checklist of items that should be included in reports of observational studies

|  | Item No | Recommendation | Page  No |
| --- | --- | --- | --- |
| **Title and abstract** | 1 | (*a*) Indicate the study’s design with a commonly used term in the title or the abstract | Title does not explicitly state design; abstract states “retrospective analysis” (Page 1, Abstract, line 1). |
|  |  | (*b*) Provide in the abstract an informative and balanced summary of what was done and what was found | Abstract includes: background, methods (121 patients, PET/MRI parameters, logistic regression, ROC, calibration, DCA), key results (IDRF, M stage, necrosis, hemorrhage, SUVmax, MTV, ADCmean, ORs, AUC=0.916, sensitivity 83.3%, specificity 81.0%), and conclusion (Page 1). |
| Introduction | | | |
| Background/rationale | 2 | Explain the scientific background and rationale for the investigation being reported | Introduction (Paragraphs 1–3): describes NB, MYCN amplification as key prognostic marker, limitations of FISH, role of PET/CT, and rationale for using PET/MRI multiparametric imaging to predict MYCN status (Pages 1–2). |
| Objectives | 3 | State specific objectives, including any prespecified hypotheses | End of Introduction: “This study integrated ¹⁸F-FDG PET/MRI-based imaging parameters and clinical data to construct a multimodal imaging model to assess the noninvasive predictive capacity for MYCN gene amplification status in high-risk neuroblastoma (HR-NB) patients.” (Page 2) |
| Methods | | | |
| Study design | 4 | Present key elements of study design early in the paper | Section 2.1, first sentence: “A retrospective analysis was conducted on the clinical and PET/MRI imaging data of 121 children with HR-NB…” (Page 2) |
| Setting | 5 | Describe the setting, locations, and relevant dates, including periods of recruitment, exposure, follow-up, and data collection | Section 2.1: “at our center between December 2018 and December 2025.” PET/MRI performed 40–60 min post-injection, before treatment. No follow-up (cross-sectional exposure/outcome assessment). (Page 2) |
| Participants | 6 | (*a*) *Cohort study*—Give the eligibility criteria, and the sources and methods of selection of participants. Describe methods of follow-up  *Case-control study*—Give the eligibility criteria, and the sources and methods of case ascertainment and control selection. Give the rationale for the choice of cases and controls  *Cross-sectional study*—Give the eligibility criteria, and the sources and methods of selection of participants | Section 2.1: inclusion criteria (1–4) and exclusion criteria (1–4). Follow-up not applicable (outcome measured at baseline). (Page 2) |
|  |  | (*b*) *Cohort study*—For matched studies, give matching criteria and number of exposed and unexposed  *Case-control study*—For matched studies, give matching criteria and the number of controls per case | Not applicable (no matching). |
| Variables | 7 | Clearly define all outcomes, exposures, predictors, potential confounders, and effect modifiers. Give diagnostic criteria, if applicable | Outcome: MYCN amplification (defined in Section 2.4 by FISH criteria). Exposures/predictors: PET/MRI parameters (SUVmax, SUVmean, SUVpeak, MTV, TLG, ADCmean, CV) and clinical variables (age, sex, site, IDRFs, INRG stage, necrosis, calcification, hemorrhage, mean diameter). Confounders not explicitly listed; multivariate regression adjusted for all variables with p<0.05 in univariate analysis. (Pages 2–3) |
| Data sources/ measurement | 8* | For each variable of interest, give sources of data and details of methods of assessment (measurement). Describe comparability of assessment methods if there is more than one group | Section 2.2 (instruments: GE SIGNA 3.0T PET/MRI), Section 2.3 (data analysis: PET VCAR software, 40% fixed-threshold for MTV, manual ROI for ADCmean, criteria for necrosis, CV formula). Same methods applied to both groups. (Pages 2–3) |
| Bias | 9 | Describe any efforts to address potential sources of bias | Section 2.6: inter-observer agreement assessed by Kappa (qualitative) and ICC (quantitative) with thresholds ≥0.75. Retrospective design acknowledged in limitations. (Page 3 and Page 8) |
| Study size | 10 | Explain how the study size was arrived at | Not explicitly justified; appears to be a convenience sample of all eligible patients during the study period (121 cases). No sample size calculation provided. (Page 2) |
| Quantitative variables | 11 | Explain how quantitative variables were handled in the analyses. If applicable, describe which groupings were chosen and why | Section 2.5: continuous variables compared using t-test or Mann-Whitney U; multivariate logistic regression with forward stepwise selection (likelihood ratio); optimal cut-offs determined by Youden index for ROC. Groupings (MNA vs MYCN-NA) based on FISH criteria (Section 2.4). (Page 3) |
| Statistical methods | 12 | (*a*) Describe all statistical methods, including those used to control for confounding | Section 2.5: logistic regression with forward stepwise selection (likelihood ratio) to adjust for confounders. Variables with p<0.05 in univariate analysis entered into multivariate model. Hosmer-Lemeshow test for model fit. (Page 3) |
|  |  | (*b*) Describe any methods used to examine subgroups and interactions | No subgroup or interaction analyses reported. |
|  |  | (*c*) Explain how missing data were addressed | No missing data mentioned; likely complete case analysis (implied by exclusion criteria). |
|  |  | (*d*) *Cohort study*—If applicable, explain how loss to follow-up was addressed  *Case-control study*—If applicable, explain how matching of cases and controls was addressed  *Cross-sectional study*—If applicable, describe analytical methods taking account of sampling strategy | Not applicable (no follow-up). |
|  |  | (*e*) Describe any sensitivity analyses | None reported. |

Continued on next page

| Results | | | |
| --- | --- | --- | --- |
| Participants | 13* | (a) Report numbers of individuals at each stage of study—eg numbers potentially eligible, examined for eligibility, confirmed eligible, included in the study, completing follow-up, and analysed | Section 3.1: 121 HR-NB patients included (49 MNA, 72 non-amplified). No flow diagram. Numbers screened/eligible/excluded not reported. (Page 3) |
|  |  | (b) Give reasons for non-participation at each stage | Not reported. |
|  |  | (c) Consider use of a flow diagram | Not provided. |
| Descriptive data | 14* | (a) Give characteristics of study participants (eg demographic, clinical, social) and information on exposures and potential confounders | Section 3.1 and Table 1, Table 2: age, sex, tumor location, IDRFs, INRG stage, necrosis, hemorrhage, mean diameter, SUVmax, SUVmean, MTV, TLG, ADCmean. (Pages 3–4) |
|  |  | (b) Indicate number of participants with missing data for each variable of interest | No missing data reported. |
|  |  | (c) *Cohort study*—Summarise follow-up time (eg, average and total amount) | Not applicable (no follow-up). |
| Outcome data | 15* | *Cohort study*—Report numbers of outcome events or summary measures over time | Section 3.1: MNA group n=49 (40.5%), MYCN-NA group n=72 (59.5%) – cross-sectional, not over time. (Page 3) |
|  |  | *Case-control study—*Report numbers in each exposure category, or summary measures of exposure |  |
|  |  | *Cross-sectional study—*Report numbers of outcome events or summary measures |  |
| Main results | 16 | (*a*) Give unadjusted estimates and, if applicable, confounder-adjusted estimates and their precision (eg, 95% confidence interval). Make clear which confounders were adjusted for and why they were included | Unadjusted: Tables 1, 2, and Section 3.3. Adjusted: multivariate logistic regression (Section 3.3) gives OR, 95% CI, p-values for SUVmax (OR=1.71), MTV (OR=1.075), ADCmean (OR=0.34). Adjusted for all variables with p<0.05 in univariate analysis (IDRFs, INRG stage, necrosis, hemorrhage, mean diameter, SUVmax, SUVmean, MTV, TLG, ADCmean). (Page 3) |
|  |  | (*b*) Report category boundaries when continuous variables were categorized | Cut-offs for SUVmax (6.07), MTV (111 cm³), ADCmean (0.678 ×10⁻³ mm²/s) given in Table 3 (derived from Youden index). (Page 5） |
|  |  | (*c*) If relevant, consider translating estimates of relative risk into absolute risk for a meaningful time period | Not applicable (cross-sectional). |
| Other analyses | 17 | Report other analyses done—eg analyses of subgroups and interactions, and sensitivity analyses | Reproducibility analyses (Section 3.5, Tables 4–5: Kappa and ICC). Calibration curves (Figure 4), DCA (Figure 3), nomogram (Figure 5). (Pages 5–8) |
| Discussion | | | |
| Key results | 18 | Summarise key results with reference to study objectives | Discussion, first paragraph: summarizes SUVmax, MTV, ADCmean as independent predictors, combined model AUC=0.916, sensitivity 0.833, specificity 0.810. (Page 6) |
| Limitations | 19 | Discuss limitations of the study, taking into account sources of potential bias or imprecision. Discuss both direction and magnitude of any potential bias | Discussion, limitations paragraph (four points): (1) limited sample size (medium-scale); (2) retrospective design and subjective interpretation; (3) single-center, equipment/regional bias; (4) lack of molecular mechanism validation. (Page 8) |
| Interpretation | 20 | Give a cautious overall interpretation of results considering objectives, limitations, multiplicity of analyses, results from similar studies, and other relevant evidence | Discussion compares with prior literature (Hu, Li, Liu, Feng, etc.), explains discrepancies (e.g., MTV/TLG significance vs. meta-analysis), highlights PET/MRI advantages, and calls for further validation. (Pages 7–8) |
| Generalisability | 21 | Discuss the generalisability (external validity) of the study results | Discussion, last paragraph: acknowledges single-center and limited sample size; calls for multicenter, large-sample prospective studies to validate and enhance generalisability. (Page 8) |
| Other information | | | |
| Funding | 22 | Give the source of funding and the role of the funders for the present study and, if applicable, for the original study on which the present article is based | Funding section: “This research was supported by the Medical Science and Technology Project of Zhejiang Province (NO. 2022KY1047, NO.2024KY1428).” Role of funders not stated. (Page 9) |

*Give information separately for cases and controls in case-control studies and, if applicable, for exposed and unexposed groups in cohort and cross-sectional studies.

**Note:** An Explanation and Elaboration article discusses each checklist item and gives methodological background and published examples of transparent reporting. The STROBE checklist is best used in conjunction with this article (freely available on the Web sites of PLoS Medicine at http://www.plosmedicine.org/, Annals of Internal Medicine at http://www.annals.org/, and Epidemiology at http://www.epidem.com/). Information on the STROBE Initiative is available at www.strobe-statement.org.
